# Electro-osmotic and viscous effects upon pressure to drive a droplet through a capillary

**DOI:** 10.1098/rspa.2021.0801

**Published:** 2022-02

**Authors:** Paul Grassia

**Affiliations:** Department of Chemical and Process Engineering, University of Strathclyde, James Weir Building, 75 Montrose Street, Glasgow G1 1XJ, UK

**Keywords:** droplet, capillary, electro-osmotic, thin film, pressure drop, waterflooding

## Abstract

A charged oil droplet advancing into a charged capillary is considered, assuming the special case in which charges are opposite and equal. The droplet is surrounded by an aqueous phase that wets the capillary wall, such that a thin film adjacent to the wall is laid down as the droplet advances. Electro-osmotic conjoining pressures contrive to make the film even thinner than in an uncharged case. The pressure drop needed to drive the droplet along is examined. The pressure drop is dominated by capillarity but contains electro-osmotic and viscous corrections. The viscous correction is shown to be remarkably insensitive to the presence of electro-osmotic effects. The electro-osmotic pressure correction is negative, reflecting work done by the electro-osmotic conjoining pressure as film is laid down. The negative electro-osmotic correction to pressure drop can far exceed the positive viscous correction. As a result, in the presence of conjoining pressures, a droplet can be driven along a capillary channel with even less pressure drop than is seen for a static uncharged droplet.

## 1. Introduction

The process of a droplet moving through a capillary is a classical problem in fluid mechanics [1,2]. Droplet motion in this context arises in many situations, e.g. in microfluidics [3–10] and flow in porous media [11–14]. Some of the main applications of interest concern using an aqueous phase that wets the capillary walls to displace a non-aqueous organic phase out of a porous medium. This sort of situation occurs in numerous applications including soil remediation [15–21] and waterflooding (or immiscible flooding more generally) for oil recovery [22–35]. In processes like these, the non-aqueous phase droplets move along capillary channels surrounded by an aqueous phase liquid. The aqueous phase is not only ahead of and behind the non-aqueous droplet but also forms a thin film between the droplet and the capillary wall. Pertinent questions [1,2] then concern what the thickness of this film might be as a function of how fast the droplet is moving and what pressure is needed to push it along, again as a function of how fast the droplet moves.

Such questions often involve more than just fluid mechanics. For example, although traditionally waterflooding has been performed with saline water, it has been found that reducing salinity levels can improve oil recovery performance [36–42], corresponding to either more flow for a given driving pressure or less driving pressure needed for a given flow. In this low salinity context, understanding waterflooding (and inter alia understanding the oil droplet motion that it induces) then involves coupling between physical chemistry and fluid mechanics. Various underlying physico-chemical mechanisms have been proposed to describe what happens in a low salinity aqueous film between a non-aqueous droplet and a capillary wall [43,44]. However, ion exchange processes (affecting surface charge adsorption at both the droplet surface and wall surface) are commonly advanced as playing a key role [38,45,46]. This in turn impacts on the thickness of the aqueous film [38,43,45] coupling back to fluid mechanics. Elucidating such mechanisms like these on the microscale is an essential step in upscaling to predict macroscale performance of low salinity waterflooding [47]. Obtaining deeper understanding of the coupled physico-chemical and fluid mechanical mechanisms and predicting what their full implications are remains pertinent, especially given that success with low salinity waterflooding on a laboratory scale does not always translate into successful field operations, as a recent review found [48].

Towards this end, the present work analyses the above-mentioned system of a moving non-aqueous droplet surrounded by an aqueous phase and a capillary wall, but allows both the droplet and the capillary wall to carry an electrical charge on their surfaces [45,49,50], a common situation for many materials when surface charge adsorption is present. Specifically, a case is considered [49,50] in which these charges are opposite and equal. The motivation for considering charged systems in general, and opposite and equal charges in particular is outlined in the next section, namely §2. This next section also identifies the specific novel contribution of the present work over and above what [45,49,50] achieved. The main theory that we will use in this work is presented in §3. Results predicted by the theory are presented in §4 and then §5 concludes the work. Mathematical details are relegated to the appendix (in the electronic supplementary material).

## 2. Background and motivation

This section reviews the important physics of droplets moving through capillary channels, starting with the uncharged case of [1,2] and continuing on to the charged cases considered by [45,49]. This then sets the context for the system to be studied in the present work, in which the droplet itself and the capillary wall carry opposite and equal charges [50]. Reasons are given why this opposite and equal charge case is of particular interest, thereby leading into the specific novel contribution of the present work, which is itself described in §2i. Readers already familiar with the work of [1,2] and with [45,49,50] may wish to skip direct to §2i. On the other hand, readers who seek more in the way of mathematical details to support the arguments given here are referred to the electronic supplementary material, appendix.

### (a) Droplet displacement: uncharged case

**Figure 1.**
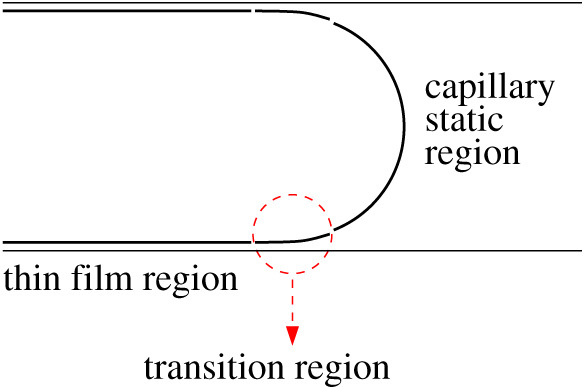
Schematic view of the front end of an advancing droplet, showing the thin film region and the capillary static region, with the transition region in between them. In this view, the droplet moves to the right, or equivalently in the frame of reference of the droplet, the capillary wall moves to the left. (Online version in colour.)

Droplet displacement problems were first tackled by [1], and the findings were as follows. A capillary number Ca can be defined (see equation (A1.1) in the electronic supplementary material, appendix) as a dimensionless measure of droplet speed, typically with Ca≪1 in problems of interest. As [1] explains, the thin film left behind as the droplet advances has a thickness order ${\mathrm{Ca}}^{2/3}$ relative to the distance across the capillary (see figure 1, which for simplicity envisages a two-dimensional system rather than an axisymmetric circular cross-section channel [51]). Capillary static regions appear at the ends of the droplet (again see figure 1, which looks specifically at the front of the droplet). The droplet is uniformly curved in these regions, and the pressure difference across the droplet interface is given by the Young–Laplace Law as a ratio between surface tension and the uniform radius of curvature.

Between the thin film and the capillary static regions, a so-called transition region appears (figure 1), in which viscous drag effects are relevant [1]. The transition region has a thickness of the order of ${\mathrm{Ca}}^{2/3}$ and a length of order ${\mathrm{Ca}}^{1/3}$, both relative to the distance across the capillary. The small thickness (remembering Ca≪1) leads to a surprisingly large shear rate and hence a surprisingly large shear stress in the transition region. When this shear stress is integrated along the transition region, what results is an order ${\mathrm{Ca}}^{2/3}$ perturbation to the pressure difference across the droplet interface, over and above the capillary static pressure [1,2,10].

Since Ca≪1 as mentioned, this ${\mathrm{Ca}}^{2/3}$ perturbation is smaller than the capillary static pressure but is nonetheless surprisingly large, given that, before analysing the problem in detail, one might anticipate instead an order Ca contribution, corresponding to viscous effects upon the pressure being linear in droplet velocity. The actual perturbation (order ${\mathrm{Ca}}^{2/3}$) is much greater than Ca when Ca is small.

The above discussion concerns just order of magnitude estimates of film thickness and pressure perturbation across the droplet. Determining these quantities more exactly requires determination of the shape of the transition region (i.e. knowledge of its thickness versus distance along it). Hence the analysis of [1] amounted to a careful study of the transition region, governing equations for which are presented in the electronic supplementary material, appendix.

Note that in general there are transition regions both at the front and the rear of the droplet, even though figure 1 shows only the front of the droplet. However, the two transition regions are slightly different in nature [1,45,52–55].

At the front of the droplet, the capillary static region is moving away from the thin film that is being laid down. At the rear of the droplet, however, the capillary static region is moving towards the thin film. A consequence of this turns out to be that, at the rear of the droplet, spatial oscillations can occur in the shape of the transition region, meaning a slightly different methodology is needed to compute it than is used at the front of the droplet [1,45,53,54]. For simplicity, we focus on the transition region at the front such as figure 1 shows. In any case, the front of the droplet must enter a capillary channel prior to the rear of the droplet doing so: computing a transition region at the front is then a necessary prerequisite of computing one at the rear [45].

### (b) Droplet displacement: charged case

All the above-mentioned results are classical fluid mechanical results, but overlook an important physico-chemical effect. Small droplets almost always carry an electric charge on their surface, as do the surfaces of the capillary walls in a porous medium. Electrical forces might therefore lead to a significant alteration of the classical theory of droplet displacement as [45] pointed out.

Such effects can be incorporated by adding an electro-osmotic tension [45,49,56,57] over and above the capillary and viscous effects that the classical theory considers. The electro-osmotic tension can also be expressed in terms of the electrical potential that develops due to the presence of charges (see [45,49] for details), but working with the electro-osmotic tension rather than with the potential turns out to be convenient. In [49], a dimensionless quantity Γ was defined (the definition is in the electronic supplementary material, appendix, see equation (A2.1)), which is the relative strength of capillary pressure and electro-osmotic tension. Electric charge effects are not relevant for large values of Γ, but might become relevant for smaller Γ values.

It was estimated by [49] (using data compiled by [45] from various sources [58–62]) that a base case value of Γ in a typical waterflooding situation might be around Γ=10. However, this relied on assuming a specific density of charged sites present on surfaces in the system, which is very sensitive to the system chemistry. As a result, it was shown by [49,50] (see also §A2 in the electronic supplementary material, appendix) that by varying the charge density, values of Γ as large as Γ=1000 (weak electro-osmotic effects only, hence close to the classical uncharged case and not requiring additional analysis) or as small as Γ=0.1 (strong electro-osmotic effects) are plausible. The present work looks specifically at Γ=10, Γ=1 and Γ=0.1.

An issue identified with using the parameter Γ however [45,49] (see also the discussion in §A2 in the electronic supplementary material, appendix) is that it only considers capillary pressure relative to a ‘bare’ electro-osmotic tension, without considering the possibility of charges being screened. In applications of interest, e.g. waterflooding for oil recovery [23], the aqueous phase is saline at least to some extent [36,39]. In line with Debye–Huckel theory for saline systems then [63,64] charge screening must take place due to electrical double layer effects, and this impacts on the electro-osmotic tension.

Another dimensionless quantity χ therefore needs to be defined [45,49] (again the definition can be found in the electronic supplementary material, appendix, equation (A2.2)). This can be viewed as the ratio between the film thickness for an advancing droplet (as predicted by the classical theory ignoring electrical charge effects) and the Debye length (which is the characteristic length scale for charge screening). Electrical charge effects were not relevant for large χ but (provided Γ itself was not too large) could become relevant for small χ.

It was found that the value of χ can be manipulated by changing the speed of the droplet [49]. Increasing the speed, i.e. increasing the capillary number, increases the predicted film thickness according to the classical theory [1], and hence increases χ. However, χ can also be manipulated by changing the salinity [49]: high salinity implies a small Debye length [63,64] and hence a large χ. The domain of χ of interest in waterflooding situations has been estimated [49,50] (using data compiled by [45] from various sources [37,38,59,62]) to be 0.02≤χ≤5. Within this domain, large χ corresponds to a high speed, high salinity waterflood whereas small χ corresponds to a low speed, low salinity waterflood (see further discussion in §A2 in the electronic supplementary material, appendix).

The impact of varying χ upon the actual film thickness (as distinct from classical film thickness ignoring electro-osmotic effects) has been considered by [49]. For a positive electro-osmotic tension (also called a ‘disjoining tension’), such that electro-osmotic effects are repulsive, the film is thicker than when such effects are absent. Repulsive electro-osmotic tensions turn out to diverge to infinity in the limit of small film thicknesses. What the film then does to prevent experiencing these diverging tensions is to thicken if χ becomes small. Essentially, the film becomes a factor up to order $\chi^{-1}$ thicker than it would be in the classical case without electro-osmotic effects [49]. Physically, this means that the film can attain a thickness comparable with the Debye length. In other words, the film can thicken to the point at which charges start to become screened.

Since film thickness (relative to the uncharged case) is found to follow the aforementioned $\chi^{-1}$ scaling very accurately, it becomes easy to extrapolate to smaller and smaller χ and still predict film thickness [49]. In fact in the small χ limit, not only did film thickness follow this $\chi^{-1}$ scaling, so did the pressure perturbation [49], i.e. the pressure driving the droplet along, over and above the leading order capillary static pressure. The reason for this is explained in the next section.

### (c) Work done to move the droplet along

Applying pressure to a moving droplet implies work is being done. At the front of the droplet, as considered here, new interface is being laid down, and much of the work is done to overcome capillary effects, i.e. at leading order, work is simply stored as interfacial energy [49]. Some additional work (an order ${\mathrm{Ca}}^{2/3}$ perturbation over and above the work at leading order) must be done to overcome viscous dissipation, specifically energy dissipated in the transition region. The above contributions to the energy both appear in the classical theory for an uncharged system however [1]. What is different in the presence of an electro-osmotic tension is that additional work must be done against that tension as new interface is laid down [49]: this is what contributes to the aforementioned $\chi^{-1}$ scaling for the pressure perturbation. This electro-osmotic work [49] (further details in the electronic supplementary material, appendix) is the integral of the electro-osmotic tension starting from a very large distance between the droplet surface and capillary wall and integrating down to whatever the eventual film thickness between the droplet surface and capillary wall turns out to be.

As mentioned above, in the presence of disjoining tension and in the small χ limit, the film thickness tends to reach a level comparable with the Debye length, i.e. the thickness above which charges start to be screened [49]. This has a number of consequences. Firstly, the film thickness is no longer sensitive to how fast the droplet is moving (i.e. no longer sensitive to capillary number) so viscous effects can be neglected in the first instance: both droplet shape and pressure drop across the droplet surface can then be obtained from an augmented Young–Laplace problem [65–68] involving just surface tension and electro-osmotic tension regardless of any relative motion.

Secondly, most of the contribution to the aforementioned ‘electro-osmotic integral’ used to compute the electro-osmotic work, might arise from just a very limited set of distances, rather than over the full integration domain. Since, in line with predictions of Debye–Huckel theory [63,64], electro-osmotic tension decays exponentially at distances far above the Debye length, it follows that large distances cannot contribute significantly to the electro-osmotic work.

Moreover, it was found [49] that if the system manages to penetrate just a little way below the Debye length, which even in the presence of disjoining tension is possible if Γ is sufficiently large (albeit not when Γ is smaller), the electro-osmotic disjoining tension begins to increase sharply. As a result, most of the electro-osmotic work is done over an interval of distances in the neighbourhood of this sharp increase in tension, the extent of this interval over which most work is done then being even rather less than the Debye length. This then helps to limit the total amount of electro-osmotic work [49]. Furthermore, distances very far below the Debye length (such that the electro-osmotic tension diverges) are invariably excluded from the integral as they are always outside the integration domain. This prevents the electro-osmotic integral (and ultimately the pressure perturbation at the front of the droplet obtained from it) from becoming even larger than the order $\chi^{-1}$ amount mentioned earlier.

### (d) Changing surface charge via charge adsorption

So far we have mentioned the role of salinity just in terms of affecting Debye length and thereby affecting χ, which recall (see §2b) is the ratio between classical film thickness and Debye length. However, the work of [45,49] indicates that salinity will have an additional role, namely it can influence net surface charge, via the mechanism of charge adsorption (explained in more detail below). The fact that salinity influences surface charge, whereas surface charge affects the process of pushing droplets surrounded by aqueous liquid along capillaries, means that there is considerable interest in using salinity as a control variable in waterflooding. Indeed, a low salinity waterflood might well behave differently from a high salinity waterflood [36–44,46–48].

The mechanism by which salinity affects waterflooding is, as alluded to above, via charge adsorption [45,49], effectively ion partitioning between bulk liquid within an aqueous film and surfaces of either a non-aqueous droplet or a capillary wall. In their ‘native’ state (assuming insignificant salinity levels and hence insignificant adsorption of charged species), both the oil droplet surface and capillary wall surface (e.g. clay surface if the capillary is found inside a porous rock) tend to be negatively charged. The density of charges on these surfaces need not be the same, being dependent upon the chemistry of each surface: in fact, it was suggested [45,49] (based on data from [60,61]) that an oil surface in its native state is likely to have a rather higher charge density than a capillary wall (i.e. clay) surface would. In a saline system, however, these negatively charged surfaces adsorb positive ions from solution [45,49], tending to cancel out part of the charge on both oil and the capillary wall.

If the saline system is multi-component containing not only monovalent positive ions (e.g. sodium) but also divalent ions (e.g. calcium), adsorption of sufficient divalent ions can cause the surface charge to switch sign. The charge adsorption mechanism thereby involves not merely ion partitioning but in fact multi-component ion exchange [38,43,45,46]. Estimates provided by [45,49] (based on data from [37,69]) suggest that clay surfaces (i.e. capillary wall surfaces in the present context) adsorb divalent ions much more readily than oil surfaces adsorb them. This means that capillary walls are very susceptible to adsorb divalent ions and change the sign of their charge from negative to positive. Oil droplets are likely however (see [45,49] based on data from [70,71]) to adsorb only monovalent ions, partially cancelling the negative charge, but not changing its sign. As mentioned above however, prior to any adsorption, oil droplets tend to have a higher charge density than capillary walls [45,49]. By adjusting the ratio between divalent to monovalent ions, and by adjusting the overall salinity, it becomes possible to find [49] sets of conditions under which the (positive) charge on the capillary wall is opposite and equal to the (negative) charge retained on the oil droplet. This then implies a significant change in the electro-osmotic behaviour as is explained next.

### (e) Droplet displacement: equal and opposite charges

For equal and opposite charges, rather than having a repulsive, disjoining electro-osmotic tension, instead an attractive, conjoining electro-osmotic pressure is present [49,50]. Consequently, rather than the film between the droplet and the capillary wall becoming thicker, instead it necessarily becomes thinner [49,50] (see also figure A1*a* in the electronic supplementary material, appendix, which plots rescaled film thicknesses in the conjoining case as computed by [50]).

Film thinning however presents its own challenges. In the repulsive, disjoining case, it was already mentioned (see §2b) that when χ≪1, the film could become a factor $\chi^{-1}$ thicker than in the classical case without electrical charge. This $\chi^{-1}$ relationship in turn makes it very simple to predict film thicknesses, which in fact now follow from an augmented Young–Laplace relationship, neglecting viscosity [49,65–68]. In an attractive, conjoining case on the other hand, we know films will become thinner, but it is unclear *a priori* how much thinner. When χ≪1, the film certainly cannot decrease in thickness by an amount $\chi^{-1}$ relative to the classical film thickness neglecting electrical charge, since that would result in a nonsense prediction of a decrease far in excess of the original classical film thickness. Moreover, trying to estimate thickness by ignoring viscosity altogether, although it might be an acceptable thing to do in the case of a disjoining film that becomes thicker, would likely be problematic in the case of a film that becomes thinner. Viscous effects might still be relevant in at least some part of the transition region if the film to which the transition region eventually attaches is especially thin.

Another key consideration in [50] concerned the functional form of the conjoining pressure with respect to distance separating the oil droplet and capillary wall (the relevant functional form is given in equation (A4.1) in the electronic supplementary material, appendix). In the limit of large distances (i.e. large separation between the droplet and the capillary wall), exponential decay is seen [49,63,66] regardless of whether disjoining or conjoining effects are present [72–74]. In the limit of small distances however, major differences occur between the disjoining and conjoining cases. Although (in the case when charges are not opposite and equal) the disjoining tension diverges in the limit of small distances (see §2b) and hence disjoining effects dominate viscous ones, in the conjoining case (when charges are opposite and equal) this does not occur. Instead, the conjoining pressure turns out to reach a finite maximum limiting value in the limit of small distances [49,50] (see §A4 in the electronic supplementary material, appendix for details). As distance reduces, the limiting value is also attained comparatively quickly in the sense that, when film thickness is small, conjoining pressure only depends on film thickness at second order. This is then an important finding since, as [49] explained, what drives fluid motion is not the conjoining pressure itself, but rather the gradient of conjoining pressure. Owing to the second-order variation, attaining the maximum conjoining pressure in the limit of a very thin film, might be associated with a small conjoining pressure gradient having a rather limited effect, by contrast with what is seen in the disjoining case.

To avoid these complications, consideration of the conjoining case was restricted by [49] to a system with a moderately large value of the parameter Γ, i.e. Γ=10: by definition (see §2b), the larger the value of Γ, the less important become electro-osmotic effects, even in situations for which the film becomes thin enough such that change in screening does not apply. In this case when Γ=10, film thickness even in the conjoining case could be readily computed as it was only ever a modest perturbation about the classical uncharged case. Even in the limit of very small χ (implying essentially no charge screening) film thickness only reduced relative to the classical case by around 10% or so [49]. Once the film thickness has been determined however, the pressure drop across the front of the droplet still needs to be computed. This is discussed next.

### (f) Work done by the electro-osmotic conjoining pressure

Despite the modest change in film thickness referred to above in the conjoining case with Γ=10, the pressure drop across the front of the droplet exhibited interesting behaviour [49]. This is still (as per §2c) a leading order capillary static pressure plus a perturbation. The perturbation moreover consists as before (again see §2c) of a viscous part plus a contribution from electro-osmotic work. The electro-osmotic work however no longer involves work done against electro-osmotic tensions. Instead, it involves work done by the electro-osmotic pressure [49] as the droplet surface approaches the capillary wall, at least at the front of the droplet where new surface is being laid down. At the rear of the droplet of course, the droplet is being peeled away from the capillary wall, but here we consider just the front. Compared with the disjoining case, here in the conjoining case, the electro-osmotic term has changed sign. For sufficiently small χ (i.e. small enough that charge screening does not apply), the work done by the electro-osmotic pressure at the front even turns out to exceed the viscous dissipation, so that the overall perturbation pressure is negative [49]. The pressure required to drive the front of the droplet along could therefore be less than the leading order capillary pressure associated with a static uncharged droplet.

It was unclear however from the data of [49] whether, in the conjoining case, the electro-osmotic work (i.e. the aforementioned ‘electro-osmotic integral’ mentioned in §2c) still exhibits a $\chi^{-1}$ scaling in the small χ limit, as happened in the disjoining case. The reason this was unclear is because with Γ=10, the electro-osmotic work (i.e. conjoining pressure integrated over distance, see the electronic supplementary material, appendix for details) remains relatively modest in the conjoining case: even though it could exceed the contribution due to viscous dissipation, in the χ domain of interest (as specified in §2b), it does not become orders of magnitude larger. In the disjoining case by contrast, the electro-osmotic work could be orders of magnitude larger [49].

Estimating the amount of electro-osmotic work done in the conjoining case is complicated further by the fact there are actually two competing effects here that influence the relative amount of electro-osmotic work done in the conjoining and disjoining cases [49]. The first effect is that (as mentioned in §2b,e) the conjoining pressure tends to remain finite whereas the disjoining tension diverges in the thin-film limit. This then tends to reduce the electro-osmotic work in the conjoining case relative to the disjoining one.

The second effect competing with this is the domain of integration, starting from a very large distance down to whatever film thickness results. In the disjoining case, as mentioned in §2b,c, there are then two possibilities. Either the final film thickness is comparable with the Debye length (so the integration never proceeds down to thicknesses where screening is removed and consequently disjoining tension never manages to increase dramatically) or else (in the event Γ increases) the system does penetrate a little way below the Debye length, in which case the majority of the electro-osmotic work is done over a small interval of distances close to the final film thickness [49]: the effective integration domain (i.e. the interval of distances over which most of the work is done) is then rather smaller than a Debye length, which again limits the total amount of electro-osmotic work.

In the conjoining case by contrast, these restrictions on the integration domain do not apply. For small χ, corresponding (as stated in §2b) to a low speed or low salinity waterflood (albeit remembering from §2d that salinity and divalent:monovalent ion ratio need to be adjusted to ensure that conditions for a conjoining pressure are met), the conjoining case can and does achieve film thicknesses far below the Debye length [49,50]. At these low film thicknesses, electro-osmotic pressure is not screened, albeit the conjoining pressure remains finite despite the lack of screening (see §2e). Distances much further away than the Debye length remain irrelevant as far as the electro-osmotic work is concerned, since electro-osmotic conjoining pressure decays exponentially there (again see §2e and in the electronic supplementary material, appendix §A4). Nevertheless, as can be seen from the material presented in the electronic supplementary material, appendix (see §A11 in particular), significant electro-osmotic work manages to be done over the full extent of the unscreened domain, starting from the Debye length down to the final film thickness. A consequence of all of this is that the electro-osmotic work in the conjoining case is potentially sensitive to the exact extent of the unscreened region over which we must integrate, meaning that it is in turn sensitive to knowing the eventual film thickness in the conjoining case, as that fixes the limit of the integration domain. The approach used by [50] to determine that film thickness is discussed next.

### (g) Finding film thickness in the conjoining case

As §2e mentioned, the work of [49] only tackled finding the film thickness in the conjoining case with Γ=10 such that film thickness was only reduced by modest amounts relative to the classical uncharged case. Cases with smaller Γ values (hence a bigger effect of conjoining pressure and consequently a more significant reduction in film thickness) were not considered by [49]. Such cases were however tackled by [50], which treated conjoining cases with Γ=1 and Γ=0.1 in addition to Γ=10. What we know of course (see §2b) is that for χ≫1, predictions of film thickness necessarily match up with the classical uncharged case. The work of [50] however managed to provide an analytic formula estimating the thickness of the thin film in the opposite limit χ≪1: the formula is given in equation (A7.1) in the electronic supplementary material, appendix .

This estimate was obtained via an analysis of the transition region that joins the thin film to a (much thicker) capillary static region. It was found [50] that the shape of the transition region could be divided into two subdomains. In the first subdomain, the transition region is much thinner than the Debye length. Although this is a subdomain in which at first sight it might appear that electro-osmotic effects matter most (being unscreened), in fact for χ≪1, they turn out to matter least. The reason is that even though the conjoining pressure is finite, its gradients are tiny, because (as mentioned earlier, see §2e) conjoining pressure has only second-order variation with film thickness in the thin film limit. Thus a capillary-viscous balance prevails as in the classical case without any electro-osmotic effects. The solution for the shape of the transition region, in this first subdomain, therefore turns out to be the same as in the classical uncharged case [1], just subjected to a rescaling to account for a much smaller overall film thickness.

In the second subdomain by contrast, the transition region has a thickness comparable with the Debye length. Viscous terms lose importance, and so the dominant balance is between the capillary and electro-osmotic terms: effectively an augmented Young–Laplace problem applies in this subdomain [49]. When the two subdomains are matched, a formula for film thickness in the thin film region, for any arbitrary Γ but in the limit as χ→0, then results (given in the electronic supplementary material, appendix as equation (A7.1) as we have said). The proposed formula was found to match well with numerical predictions [50] in the cases Γ=10 and Γ=1 (see figure A1*a* in the electronic supplementary material, appendix).

In addition to this, an approximate formula for how film thickness varies with χ (again for any arbitrary Γ) interpolating between the χ≪1 and χ≫1 behaviours was also proposed [50]. Again this is given in the electronic supplementary material, appendix (equation (A8.1)). This was based on only an ad hoc approximation, so was less reliable than either of the predictions in the χ≪1 and χ≫1 limits. Nevertheless, it was still borne out reasonably well in the Γ=10 and Γ=1 cases studied by [50]. However, the Γ=0.1 case also studied by [50] proves to be more complicated as is discussed next.

### (h) Multiple solution branches

When the transition region at the front of the droplet was analysed in the conjoining case with Γ=0.1, there turned out to be multiple solution branches, at least three of them [50]. Physically this means that, at any specified χ, there are multiple possible solutions for film thickness for a droplet advancing at a certain speed (see figure A1*b* in the electronic supplementary material, appendix).

The three solution branches can be labelled upper (the one with the highest thickness in the thin film region), lower (the one with the lowest film thickness) and intermediate (a film thickness in between the two). It is the upper solution branch that joins up with the classical uncharged solution expected in the χ≫1 limit [50]. In this limit, charges are strongly screened, so the classical uncharged solution is recovered. It is the lower solution branch however that joins up with the film thickness formula proposed in the χ→0 limit [50].

Examination of the shape of the transition region at the front of the droplet revealed the difference between the lower and upper branches [50]. For the lower branch, the thickness of the transition region was found to grow monotonically from the thin film region to the capillary static region. In the case of the upper branch however, as the value of χ decreased, the transition region was shown to exhibit a spatial oscillation. Specifically moving away from the thin film region, the transition region thickness was found to grow to a local maximum, then it falls to a local minimum (albeit not quite so thin as the thin film region), then grows monotonically after that.

Such oscillations were shown by [50] to be associated with the gradient of capillary pressure offsetting the gradient of the electro-osmotic pressure. The oscillations in the transition region at the front of the droplet are of interest because they only occur in the case of an electro-osmotic conjoining pressure. Neither the classical case (without electric charge) nor the case with an electro-osmotic disjoining tension exhibit oscillations at the front of the droplet. The classical case does admit oscillations but only the rear of the droplet (see §2a and also references cited therein [45,53,54]), and these oscillations turn out moreover to be eliminated by a sufficiently strong disjoining tension as [49] found. As mentioned therefore, oscillations at the front of the droplet are specific to the conjoining case under discussion here.

So far we have discussed only the upper and lower solution branches, but not the intermediate branch. The intermediate branch was found by [50] to be a solution that ‘dithers’ forming a boundary between very slow monotonic growth requiring a very long distance to be realized, or an oscillation with a very long wavelength, again requiring a very long distance to be observed. The intermediate solution branch is therefore inherently difficult to calculate (see the electronic supplementary material, appendix §§A6 and A9 for more details).

It is unclear whether the intermediate branch solution is even physically relevant at all: the entire basis for computing the transition region [1] relies on this region being a small fraction of the overall length of the droplet. If however the solution for the transition region extends over too large a spatial distance [50], the basis upon which the solution has been obtained is no longer valid. Indeed, it is possible that the intermediate solution branch is not even a stable solution branch. The solution technique used by [50] for identifying the solution branches (see also §A5 in the electronic supplementary material, appendix) involves a steady-state analysis in the reference frame of the droplet and does not interrogate the stability of those steady solutions to small perturbations.

### (i) Novel contribution of the present work

To summarize, the work of [50] identified solutions for the transition region and also determined the thickness of the thin film region, in situations in which significant electro-osmotic conjoining pressures were present (small Γ) with films thin enough to prevent charge screening (small χ). What the previous work did not achieve however was to compute the pressure difference at the front of the droplet associated with each film thickness computed. More specifically, it did not compute the perturbation to the pressure difference across the droplet in the small Γ and small χ limit, remembering that pressure difference involves a leading order capillary static term plus a perturbation involving viscous and electro-osmotic contributions, the latter contribution (according to §2f) being negative in the conjoining case. Computing and analysing these perturbations to the pressure difference is the novel contribution that the present work achieves.

Specifically, we will demonstrate that, even in the conjoining case, the electro-osmotic contribution to the pressure does indeed exhibit a $\chi^{-1}$ scaling in the small χ limit, despite this having been inconclusive in the data of [49]. Compared with [49], smaller Γ values are considered here, enhancing the importance of these electro-osmotic contributions. We will however explore in addition how the viscous contribution to the pressure difference behaves as a function of χ. We will find that this viscous contribution is surprisingly insensitive to film thickness and hence surprisingly insensitive to χ, despite χ having a major impact on film thickness.

## 3. Theory

The main theoretical result presented in this work is an expression (equation (3.1) below) for the pressure drop across the front of a droplet advancing into a capillary as per figure 1. The derivation of this result is in the electronic supplementary material, appendix, building on the work of [49,50]. As discussed in the electronic supplementary material, appendix, we employ dimensionless variables with pressure made dimensionless on a capillary pressure scale. At leading order, the dimensionless pressure drop across the front of the droplet turns out to be unity. Physically this has a simple interpretation, already alluded to in §2c: the work that is required to push the front of the droplet along must match the capillary surface energy associated with new surface created at the front of the droplet as it advances.

This is the leading order balance, but (as mentioned in §2a,c,f) there are perturbation corrections to the pressure. The dimensionless pressure perturbation can be written as ${\left(3\mathrm{Ca}\right)}^{2/3}\Delta p^{*}$, where Ca is (as already mentioned) the capillary number, and where $\Delta p^{*}$ is a value that we must compute. In the electronic supplementary material, appendix it is shown (see §A10) that $\Delta p^{*}$ can be written as

3.1 $$\Delta p^{*}=J^{*}+V+I_{\mathrm{EO}}.$$

Here, $J^{*}$ is a geometric pressure correction, V is a viscous dissipation pressure correction and $I_{\mathrm{EO}}$ is an electro-osmotic pressure correction. Expressions for these quantities are obtained in the electronic supplementary material, appendix. In §3a–c below, we describe what the terms represent physically.

### (a) Geometric pressure correction

The geometric correction in equation (3.1) comes about for the following reason. Even though, as mentioned previously, the leading order energy balance in the system is between the work done by the pressure drop and capillary surface energy created at the front, in fact work is only done across that part of the channel that is actually filled by the droplet. It turns out (see §A3 in the electronic supplementary material, appendix) that a thin film of thickness $h^{*}={\left(3\mathrm{Ca}\right)}^{2/3}J^{*}$ is left behind as the droplet advances. Here, $h^{*}$ is made dimensionless relative to the half-thickness of the channel, this half-thickness being taken as the unit of length (recalling from §2a that figure 1 is considered to represent a two-dimensional system, rather than an axisymmetric one). Meanwhile $J^{*}$ is a quantity that must be determined (figure A1). Given that film is left behind as the droplet advances, the droplet almost, but not quite, fills the channel. To compensate, the driving pressure drop therefore needs to be slightly higher by this same amount to ensure that the required amount of work is done.

### (b) Viscous pressure correction

The viscous contribution V in equation (3.1) is easy to understand. If the droplet advances at a known rate, there is a well-defined rate of working by the pressure, but also a well-defined viscous energy dissipation rate, with dissipation here occurring in the transition region. The pressure drop at the front of the droplet therefore needs to increase to overcome the viscous dissipation and this is what V quantifies. Given that dissipation is associated with the transition region, in cases that admit multiple solution branches (see §2h), the most dissipative branches tend to be those that extend the transition region spatially (as §A12 in the electronic supplementary material, appendix explains).

### (c) Electro-osmotic pressure correction

Both terms $J^{*}$ and V occur in the classical uncharged system [1], although their values can be affected in the presence of surface charge. In a charged system however there is another contribution to $\Delta p^{*}$ namely the term $I_{\mathrm{EO}}$, which is an electro-osmotic work integral [49], mentioned in §2c,f (see §A11 in the electronic supplementary material, appendix for details). Physically, $I_{\mathrm{EO}}$ arises as follows. As new film is laid down immediately behind the capillary static region, it is initially comparatively thick. However, as the front of the droplet moves further and further ahead of a fixed location in the capillary channel, the film at that location becomes thinner. As discussed in §2c,f, work is then either done against the charges (a disjoining/repulsive case) or else by the charges (a conjoining/attractive case), and this leads to $I_{\mathrm{EO}}$.

Although $J^{*}$ and V are always positive, in the conjoining case, $I_{\mathrm{EO}}$ is negative. Hence in this case $\Delta p^{*}$ may be either positive or negative [49], depending on which term dominates equation (3.1): this is what we explore in the next section. Note however that in the conjoining case, the film invariably ends up thinner than in the classical uncharged case. Hence $J^{*}$ decreases being just a measure of the film thickness ($h^{*}={\left(3Ca\right)}^{2/3}J^{*}$). It then follows that the geometric contribution in equation (3.1) is less important than the viscous and electro-osmotic contributions. Hence much of the focus in the section to follow will be on these latter two terms.

## 4. Results

This results section is laid out as follows. In §4a, data for the pressure drop are considered for various values of the parameters Γ and χ. Then in §4b, data for the electro-osmotic work integral are examined. Finally, in §4c, the contribution of viscous dissipation is considered.

### (a) Pressure drop correction

**Figure 2.**
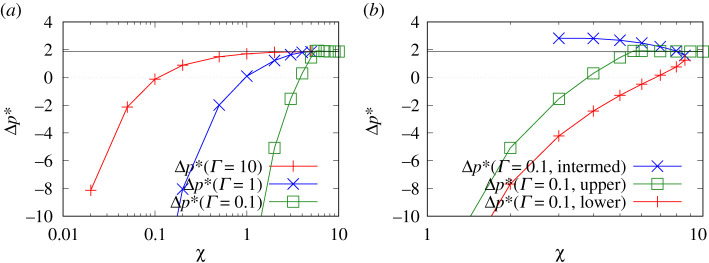
(*a*) $\Delta p^{*}$ versus χ for different values of Γ, either 10, 1 or 0.1. In the case of Γ=0.1, only the upper solution branch is shown. (*b*) $\Delta p^{*}$ versus χ for Γ=0.1 showing all three solution branches. In both (*a*) and (*b*), the horizontal line corresponds to the classical uncharged case. (Online version in colour.)

Figure 2*a* presents data for how the correction to the pressure drop across the front of the droplet $\Delta p^{*}$ varies with χ for various Γ, namely Γ=10, Γ=1 and Γ=0.1. At each Γ, for sufficiently large χ, the $\Delta p^{*}$ value approaches the classical uncharged value [1], which we denote $\Delta p_{\mathrm{Breth}}^{*}$ (the subscript refers to the author of [1]). As mentioned in §2b, the domain of χ of interest here is between χ=0.02 (typical of a low speed, low salinity waterflood) and χ=5 (typical of a high speed, high salinity waterflood). In the case Γ=0.1, we have however extended the domain even further up to χ=10, in order to make it more evident how the solution matches up with $\Delta p_{\mathrm{Breth}}^{*}$ in the large χ limit. Note though that for Γ=0.1, there are three solution branches but figure 2*a* only plots the upper branch as this is the one for which $\Delta p^{*}$ joins up with $\Delta p_{\mathrm{Breth}}^{*}$.

What is clear in figure 2*a* is that as χ falls, the value of $\Delta p^{*}$ switches from being positive to being negative, meaning that it is easier (i.e. less pressure is required) to push the front of the droplet along. As already mentioned (see §2f), this is due to electro-osmotic work being done by the system, the electro-osmotic work integral $I_{\mathrm{EO}}$ being the only negative contribution to $\Delta p^{*}$. The electro-osmotic work now exceeds the energy lost to viscous dissipation, although it is worth remembering that the leading order contribution to the pressure drop is always a capillary term, whereas the various terms being considered here are just perturbations.

When Γ=10, a substantial decrease in χ is needed for $\Delta p^{*}$ to switch sign from positive to negative. Indeed when we reach χ=0.02 (the smallest χ considered here), the $\Delta p^{*}$ value, while it has switched sign relative to $\Delta p_{\mathrm{Breth}}^{*}$, is not yet orders of magnitude larger than $\Delta p_{\mathrm{Breth}}^{*}$, a point already noted by [49]. In the cases Γ=1 and Γ=0.1 however, decreasing χ causes $\Delta p^{*}$ to switch sign sooner. In these cases, values of $\Delta p^{*}$ for χ=0.02 are outside the scale of the plot in figure 2*a*, but turn out to be orders of magnitude larger than (and opposite sign from) $\Delta p_{\mathrm{Breth}}^{*}$.

Figure 2*b* shows the case Γ=0.1 but as there are now three solution branches, data for all three of them are plotted here. Both the upper solution branch and the lower solution branch switch $\Delta p^{*}$ from being positive to being negative as χ decreases. However, the lower solution branch $\Delta p^{*}$ switches sign sooner, and is consistently more negative than the upper branch $\Delta p^{*}$.

This is unsurprising since the lower solution branch corresponds to a thinner film (i.e. a lower $J^{*}$) than the upper branch does, see e.g. Figure A1*b* in the electronic supplementary material, appendix. The negative $\Delta p^{*}$ ultimately arises from the electro-osmotic work $I_{\mathrm{EO}}$, and the thinner the film becomes eventually, the more electro-osmotic work is done by the conjoining pressure starting from a very large thickness down to that thinner film. The more negative $\Delta p^{*}$ could also be associated with less viscous dissipation in the lower branch than in the upper branch, a point we revisit in §4c.

Despite the quantitative difference in $\Delta p^{*}$ between the lower and upper branches, figure 2*b* shows that qualitatively both branches exhibit the same trend. The intermediate branch however behaves differently. Rather than $\Delta p^{*}$ decreasing and eventually becoming negative as χ decreases, instead $\Delta p^{*}$ grows with decreasing χ, at least over the domain of χ plotted here: for reasons explained in the electronic supplementary material, appendix (see §§A6 and A9), data for any smaller χ are inherently difficult to calculate for this branch. Since the dominant positive contribution to $\Delta p^{*}$ within equation (3.1) is expected to come from the viscous dissipation term V, the intermediate branch appears to be substantially more dissipative than the other two branches are, again a point to be revisited in §4c. Before considering viscous dissipation however, we first consider electro-osmotic work. This is defined formally in equation (A10.9), and is analysed in detail in §A11 in the electronic supplementary material, appendix: the results presented in what follows draw upon that analysis.

### (b) Electro-osmotic work integral

**Figure 3.**
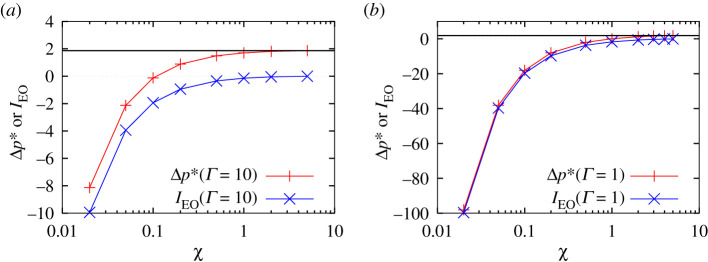
(*a*) $\Delta p^{*}$ versus χ compared with $I_{\mathrm{EO}}$ vs χ in the case (*a*) Γ=10 and (*b*) Γ=1. The horizontal line corresponds to the classical uncharged case $\Delta p_{\mathrm{Breth}}^{*}$. (Online version in colour.)

Figure 3*a* returns to consider the case Γ=10 comparing now $\Delta p^{*}$ with the electro-osmotic work integral $I_{\mathrm{EO}}$ given by equation (A10.9). Since $\Delta p^{*}$ eventually turns negative at small enough χ, and since $I_{\mathrm{EO}}$ is the only negative contribution to $\Delta p^{*}$, it follows that $I_{\mathrm{EO}}$ becomes the dominant contribution to $\Delta p^{*}$ when χ is small. Figure 3*b* shows an analogous plot but for Γ=1 instead of Γ=10. On the scale of this plot, it is difficult to see the difference between $\Delta p^{*}$ and $I_{\mathrm{EO}}$. Indeed, $I_{\mathrm{EO}}$ is very strongly the dominant contribution to $\Delta p^{*}$ when χ is small.

The increased importance of $I_{\mathrm{EO}}$ for decreases in both Γ and χ can be seen by consulting a simple asymptotic expression $I_{\mathrm{EO},\mathrm{asymp}}$ (i.e. $I_{\mathrm{EO},\mathrm{asymp}}\equiv -2/(\Gamma \chi )$, see equation (A11.4) in §A11 of the electronic supplementary material, appendix), which scales inversely with both these parameters. Note the physical difference between $I_{\mathrm{EO}}$ and $I_{\mathrm{EO},\mathrm{asymp}}$. The former represents electro-osmotic work done between an arbitrarily large thickness and some eventual film thickness, whereas the latter represents electro-osmotic work done between an arbitrarily large and an arbitrarily small thickness. For a sufficiently thin film however, the two expressions should be comparable.

**Figure 4.**
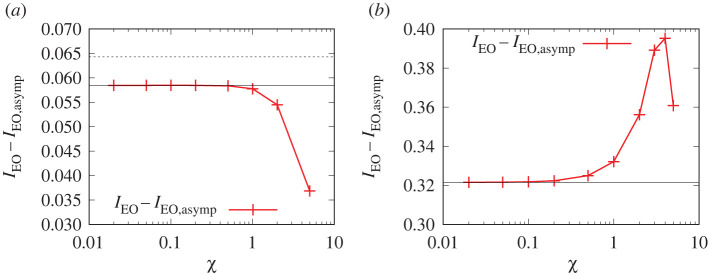
$I_{\mathrm{EO}}-I_{\mathrm{EO},\mathrm{asymp}}$ versus χ for (*a*) Γ=10 and (*b*) Γ=1. In both cases, the horizontal line corresponds to $J_{\mathrm{Breth}}^{*}/(\Gamma (1+1/\Gamma ))$, which is the χ→0 limiting value of $I_{\mathrm{EO}}-I_{\mathrm{EO},\mathrm{asymp}}$. For sufficiently large Γ, this approximates to $J_{\mathrm{Breth}}^{*}/\Gamma$ (the dashed line in (*a*)). (Online version in colour.)

**Figure 5.**
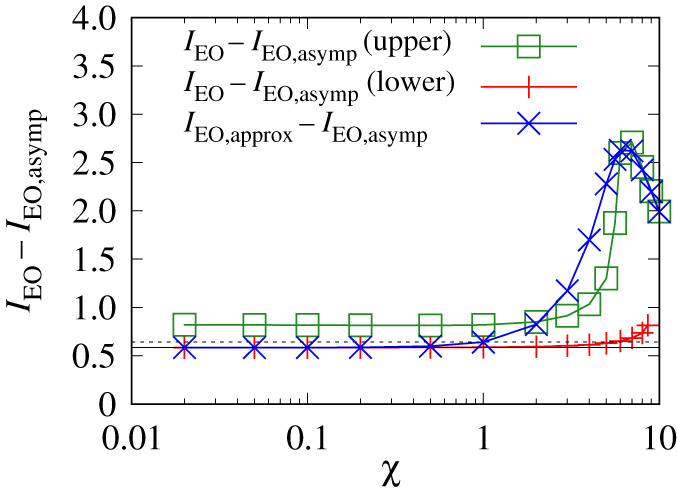
$I_{\mathrm{EO}}-I_{\mathrm{EO},\mathrm{asymp}}$ versus χ for Γ=0.1 for the upper and lower solution branches. Also shown is an approximation $I_{\mathrm{EO},\mathrm{approx}}-I_{\mathrm{EO},\mathrm{asymp}}$ that is obtained by using an approximate formula for $J^{*}$ versus χ which interpolates between the small χ and large χ asymptotic behaviour of $J^{*}$. The horizontal line is $J_{\mathrm{Breth}}^{*}/(\Gamma (1+1/\Gamma ))$, which is the χ→0 limiting value of $I_{\mathrm{EO}}-I_{\mathrm{EO},\mathrm{asymp}}$. For sufficiently small Γ, this approximates to $J_{\mathrm{Breth}}^{*}$ (dashed line). (Online version in colour.)

To summarize, understanding how $\Delta p^{*}$ behaves with variations in Γ and χ is closely connected with how $I_{\mathrm{EO}}$ behaves. The value of $I_{\mathrm{EO}}$ in turn can be written as the sum of a dominant term $I_{\mathrm{EO},\mathrm{asymp}}$ and a correction $I_{\mathrm{EO}}-I_{\mathrm{EO},\mathrm{asymp}}$. The behaviour of $I_{\mathrm{EO},\mathrm{asymp}}$ is however given straightforwardly by equation (A11.4). Hence, we focus in what follows specifically on the correction $I_{\mathrm{EO}}-I_{\mathrm{EO},\mathrm{asymp}}$. This is plotted in figures 4 and 5.

At sufficiently large χ it is known from §A11 in the electronic supplementary material, appendix (see equation (A11.3)) that $I_{\mathrm{EO}}$ decays exponentially, meaning that $I_{\mathrm{EO}}-I_{\mathrm{EO},\mathrm{asymp}}$ becomes effectively $-I_{\mathrm{EO},\mathrm{asymp}}$, which via equation (A11.4) decays at large χ albeit algebraically rather than exponentially. At sufficiently small χ, the value of $I_{\mathrm{EO}}-I_{\mathrm{EO},\mathrm{asymp}}$ reaches a limiting value, which equation (A11.7) estimates to be $J^{*}/\Gamma$, where recall $J^{*}$ is a rescaled film thickness (see e.g. §3a). In the χ→0 limit, however (as already alluded to in §2g), we have a simple expression for $J^{*}$ given by equation (A7.1). Specifically, $J^{*}{|}_{\chi \to 0}=J_{\mathrm{Breth}}^{*}/(1+1/\Gamma )$, where $J_{\mathrm{Breth}}^{*}$ denotes the classical uncharged value from [1]. Thus $I_{\mathrm{EO}}-I_{\mathrm{EO},\mathrm{asymp}}$ is found to reduce to $J_{\mathrm{Breth}}^{*}/(\Gamma (1+1/\Gamma ))$. For Γ=10, this is relatively close to $J_{\mathrm{Breth}}^{*}/\Gamma$ as figure 4*a* shows.

Note that as χ increases from zero, the value of $J^{*}$ also increases above the value predicted by equation (A7.1). Hence $I_{\mathrm{EO}}-I_{\mathrm{EO},\mathrm{asymp}}$ can also increase, since as mentioned, according to equation (A11.7) this is well approximated by $J^{*}/\Gamma$ (this applies provided $J^{*}\chi \ll 1$). In the case Γ=10, an increase in $I_{\mathrm{EO}}-I_{\mathrm{EO},\mathrm{asymp}}$ is difficult to see however, because there are only very modest increases in $J^{*}$ with increasing χ (see also figure A1*a*). For Γ=1, more substantial increases in $J^{*}$ (see figure A1*a*) and hence in $I_{\mathrm{EO}}-I_{\mathrm{EO},\mathrm{asymp}}$ are possible, as is evident in figure 4*b*. Of course, for sufficiently large χ, the value of $I_{\mathrm{EO}}-I_{\mathrm{EO},\mathrm{asymp}}$ decreases again for reasons already explained, i.e. $I_{\mathrm{EO}}$ decays exponentially while $I_{\mathrm{EO},\mathrm{asymp}}$ obeys equation (A11.4).

The value of $I_{\mathrm{EO}}-I_{\mathrm{EO},\mathrm{asymp}}$ for Γ=0.1 is shown in figure 5. We show the upper and lower branches only. The intermediate branch (not shown here) would give $I_{\mathrm{EO}}-I_{\mathrm{EO},\mathrm{asymp}}$ between the upper and lower branch values. However, knowledge of $I_{\mathrm{EO}}$ is of limited use for the intermediate branch, since it has little correlation with how $\Delta p^{*}$ behaves (figure 2*b*).

Figure 5 shows that $I_{\mathrm{EO}}-I_{\mathrm{EO},\mathrm{asymp}}$ for the upper branch has a significant increase as χ increases. This again follows from equation (A11.7), namely $I_{\mathrm{EO}}-I_{\mathrm{EO},\mathrm{asymp}}\approx J^{*}/\Gamma$, with $J^{*}$ undergoing a significant increase with increasing χ (see figure A1*a*). At large χ, the upper branch $I_{\mathrm{EO}}-I_{\mathrm{EO},\mathrm{asymp}}$ decays in figure 5: $J^{*}\chi$ is now large, so equation (A11.7) does not apply.

Unlike the upper branch, the lower branch in figure 5 shows only a modest increase in the value of $I_{\mathrm{EO}}-I_{\mathrm{EO},\mathrm{asymp}}$ as χ increases. This is as expected based on equation (A11.7), since the lower branch $J^{*}$ itself has only a modest increase (figure A1*b*). Note also that the lower branch $I_{\mathrm{EO}}-I_{\mathrm{EO},\mathrm{asymp}}$ is smaller than the upper branch one, which follows via equation (A11.7) since the lower branch $J^{*}$ is also smaller. As χ→0, as mentioned earlier, $I_{\mathrm{EO}}-I_{\mathrm{EO},\mathrm{asymp}}$ reduces to $J_{\mathrm{Breth}}^{*}/(\Gamma (1+1/\Gamma ))$. When Γ is small (e.g. Γ=0.1) this in turn is close to $J_{\mathrm{Breth}}^{*}$ as figure 5 shows. Also plotted in figure 5 is an approximate formula $I_{\mathrm{EO},\mathrm{approx}}-I_{\mathrm{EO},\mathrm{asymp}}$ (see equation (A11.9)). This employs an *ad hoc* interpolation formula for film thickness (mentioned in §2g; see also equation (A8.1)). In figure 5, this matches the upper branch result when χ is large, but transitions to the lower branch result when χ is small.

### (c) Viscous dissipation

The previous section analysed the behaviour of $I_{\mathrm{EO}}$, which is the main contribution to $\Delta p^{*}$ when Γ and χ are small. However, according to equation (3.1), $\Delta p^{*}$ also has a geometric contribution $J^{*}$ and a viscous contribution V. We now examine how these terms behave, drawing also upon an analysis presented in §A12 in the electronic supplementary material, appendix.

**Figure 6.**
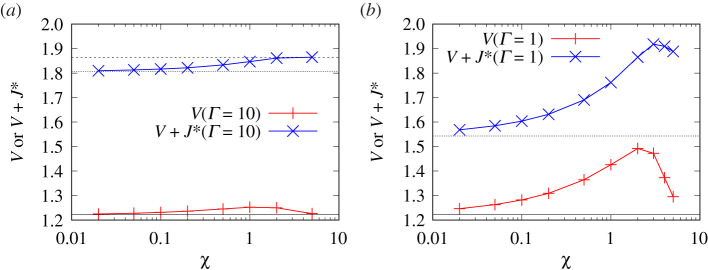
V and/or $V+J^{*}$ versus χ for (*a*) Γ=10 and (*b*) Γ=1. The horizontal solid line indicates $V_{\mathrm{Breth}}$. The horizontal long dashed line indicates $\Delta p_{\mathrm{Breth}}^{*}=V_{\mathrm{Breth}}+J_{\mathrm{Breth}}^{*}$. The horizontal short dashed line indicates $V_{\mathrm{Breth}}+J^{*}{|}_{\chi \to 0}$. (Online version in colour.)

Figure 6 plots both $V+J^{*}$ and V as functions of χ. The Γ values considered are Γ=10 and Γ=1. Data for both Γ values exhibit qualitatively similar behaviour. In the limit χ≫1, the sum $V+J^{*}$ approaches the classical uncharged $\Delta p_{\mathrm{Breth}}^{*}$. As χ begins to decrease, we see at first limited changes in $V+J^{*}$. Although $J^{*}$ is decreasing, it turns out that V increases, and these changes roughly cancel each other out. As more substantial decreases in χ occur however, $J^{*}$ falls from $J_{\mathrm{Breth}}^{*}$ to a lesser value $J^{*}{|}_{\chi \to 0}=J_{\mathrm{Breth}}^{*}/(1+1/\Gamma )$, as given by equation (A7.1) in the electronic supplementary material, appendix. Decreases in $V+J^{*}$ are then seen, and are almost entirely due to the decrease in $J^{*}$.

Indeed, the value of V as χ→0 is almost the same as the classical uncharged value (denoted here $V_{\mathrm{Breth}}$) that is realized when χ≫1. This result follows from reasons explained in §A12 in the electronic supplementary material, appendix: when χ≪1, the transition region can be divided (as mentioned already in §2g) into a subdomain in which a classical capillary-viscous balance applies and another subdomain with a capillary-electro-osmotic balance. As §A12 indicates, the contribution to V comes almost entirely from the first subdomain and so agrees with $V_{\mathrm{Breth}}$. This can happen despite both the film and transition region becoming thinner when χ is smaller (thereby leading to a higher shear rate and more viscous shear stress, and consequently more energy dissipation in any given fluid volume). Nevertheless, the value of V changes little from $V_{\mathrm{Breth}}$ because (as §A12 further explains) the size of the transition region also scales to compensate.

**Figure 7.**
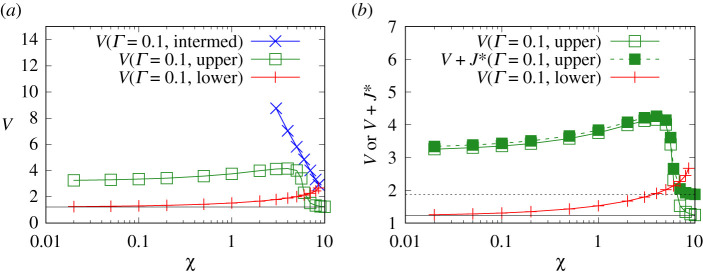
(*a*) V versus χ for Γ=0.1. Three solution branches (intermediate, upper and lower) are shown. The horizontal solid line indicates $V_{\mathrm{Breth}}$. (*b*) V and/or $V+J^{*}$ versus χ for Γ=0.1. Only the upper and lower branches are shown. The horizontal solid line indicates $V_{\mathrm{Breth}}$. The horizontal long dashed line indicates $\Delta p_{\mathrm{Breth}}^{*}=V_{\mathrm{Breth}}+J_{\mathrm{Breth}}^{*}$. (Online version in colour.)

Figure 7*a* considers V values for a system with a smaller Γ value, namely Γ=0.1. Here, there are three solution branches. We see that the intermediate solution branch is the most dissipative of all. This aligns with the arguments presented in §A12. The intermediate branch is (as mentioned in §2h) a ‘dithering’ solution that requires a very long distance to experience a definitive growth in the thickness of the transition region, and which §A12 argues is therefore dissipative over a very long distance.

The next most dissipative branch is by and large, the upper branch. Recall (see §2h) that this branch exhibits a spatial oscillation in the thickness of the transition region, which extends the spatial region over which dissipation occurs, compared with the lower branch which exhibits no oscillation. Admittedly, there is a region in figure 7 with χ values greater than about 6, in which the upper branch appears to be less dissipative than the lower branch. However, as already explained (see §§2b and 4a) the domain of interest is only up to χ of around 5, which corresponds to a high speed, high-salinity waterflood. The reason why figure 7 has been continued to χ>5 is to show how (even with Γ as small as Γ=0.1) the upper branch eventually joins up with the classical uncharged solution at large enough χ. Given that the classical uncharged transition region is non-oscillatory, this means that for large enough χ, oscillations disappear from the upper branch too: as a consequence, the spatial extension of the transition region is less, and the dissipation is less also. For χ≤5, however (which is the domain of interest here), the upper branch definitely has a spatial oscillation in the shape of the transition region (as is clear from data in [50]) and so is more dissipative than the lower branch is.

A zoomed view for the lower and upper branches is shown in figure 7*b*. For the upper branch, values of both V and $V+J^{*}$ are shown. Significant differences between V and $V+J^{*}$ are only really seen at large χ values, since decreasing χ causes $J^{*}$ to fall. In the case of the lower branch, only the value of V (and not the value of $V+J^{*}$) is plotted. This is because $J^{*}$ tends to be rather small along the entire lower branch, so distinguishing V and $V+J^{*}$ would be difficult on the scale of the graph. It is clear that for small χ, the lower branch has considerably smaller V than the upper branch at the same χ, and moreover the χ→0 limit of V on the lower branch matches the χ≫1 limit of V on the upper branch (i.e. the classical uncharged value $V_{\mathrm{Breth}}$).

## 5. Conclusion

In the context of e.g. waterflooding for oil recovery [23], we have considered a charged oil droplet advancing into a charged capillary [45], in a special case with opposite and equal charges on the droplet surface and the capillary wall. The oil droplet is surrounded by an aqueous layer that wets the capillary wall, and the opposite and equal charge state can in principle be achieved by manipulating the salinity as well as the ratio of divalent to monovalent ions in the aqueous layer [49,50]. Electro-osmotic conjoining pressures are then present and cause the aqueous film that is left behind as the droplet advances to be thinner (and possibly much thinner) than in the case without electrical charge [1] and/or a charged case with electro-osmotic disjoining tensions [45,49].

The relative importance of the electro-osmotic conjoining pressure [50] is governed by two dimensionless parameters Γ (the ratio between capillary and electro-osmotic pressures) and χ (the extent to which electro-osmotic effects are screened, given by a ratio between a classical film thickness without electro-osmotic effects and the Debye length): decreasing either of these parameters increases the importance of electro-osmotic conjoining effects. Physically decreasing Γ would be achieved by having a higher density of adsorption sites on the oil droplet and capillary wall. Decreasing χ is achieved by reducing droplet speed (which reduces film thickness even in the absence of electro-osmotic effects) or decreasing salinity (which increases Debye length).

In addition to computing film thickness as a function of Γ and χ, we can also obtain pressure drop across the front of the droplet as a function of these variables. This consists of a leading order capillary static pressure plus perturbation corrections. The novel aspect of this work has been to examine these pressure corrections in detail and findings are outlined in §5a,b below.

### (a) Relative importance of pressure drop contributions

The perturbation correction to the pressure drop across the droplet is comprised of three terms: a geometric, a viscous and an electro-osmotic term. The first term (i.e. the geometric correction) arises because the work done by the applied pressure to advance the droplet is not done across the full width of the capillary channel, but instead only across that part of the width occupied by the droplet. Pressure needs to be increased slightly to compensate for work being done across not quite the full channel width. This geometric term turns out to be unimportant in the conjoining case, because the film becomes very thin indeed, so the amount of geometric correction is tiny.

The second term, namely, the viscous correction to the pressure, is associated with the rate of viscous dissipation. This turned out to be surprisingly insensitive to the electro-osmotic conjoining effects. The result is counterintuitive because thinning a film and thereby thinning the transition region to which it is attached increases both the shear rate and shear stress in this region, which then in principle can influence viscous dissipation. The overall viscous dissipation rate can remain unaffected however because the transition region shrinks in size both across the thickness of the capillary channel and along the capillary wall and this compensates. Moreover, when the transition region is rescaled to account for the shrinking of these length scales, its shape (or at any rate its shape in a subdomain towards its thinner end) is nearly the same regardless of whether or not electro-osmotic effects are present. The reason is that even though electro-osmotic conjoining pressures can be significant in these thinner parts of the transition region, the gradients of those conjoining pressures remain small. It is the gradient of the conjoining pressure rather than the conjoining pressure itself that governs the shape of the transition region. Hence an uncharged case and a case with strong conjoining effects predict a surprisingly similar shape for the transition region and hence for the viscous contribution to pressure drop that depends on that shape.

It is only when multiple solution branches occur (the small Γ limit) that we see variation of the viscous contribution to pressure drop which becomes sensitive to the particular solution branch. This comes about because different branches have different shapes for the transition region as seen in [50]. The lower branch exhibits a monotonic increase in the thickness of the transition region but the upper branch exhibits a spatial oscillation with monotonic increase thereafter. Owing to this oscillation, the upper branch maintains the transition region thickness comparatively small over a longer spatial distance, so tends be more dissipative, i.e. it has a larger viscous contribution to the pressure drop. The intermediate branch is even more dissipative. This solution branch ‘dithers’ as [50] found, so any definitive increase in the thickness of the transition region is delayed over a very large spatial distance, which leads to considerable dissipation.

The third contribution to the perturbation to the pressure drop across the front of the droplet is an electro-osmotic integral [49]. In the limit of small Γ and small χ, this is by far the most important contribution to the pressure drop perturbation, scaling inversely proportional to both Γ and χ. In the conjoining case considered here, this is moreover a negative contribution to the pressure drop: the droplet surface and capillary wall are attracted to each other so work is done by the conjoining pressure as new film is laid down. For small Γ and χ, the overall pressure drop is then comprised of a leading order capillary static pressure plus a negative perturbation term. This is then less than the pressure drop across an uncharged droplet, even a perfectly stationary one.

This contrasts with the case of a repulsive disjoining tension [49], for which work must be done against that tension, such that pressure drop needed to drive the front of the droplet along could increase considerably. In this case, tensions would become very large at small film thicknesses and so films thicken to prevent large tensions from appearing. This limits the amount of electro-osmotic work done as additional film is laid down as the front of the droplet advances: work is only done starting from very large distances down to whatever eventual film thickness results. Moreover, even if tensions do increase markedly close to that final film thickness, the increase tends to be confined to just a very limited spatial region, which again limits the electro-osmotic work.

In the conjoining case considered here by contrast, conjoining pressures remain finite at very small thicknesses. In spite of this, a significant amount of work (scaling like ${\left(\Gamma \chi \right)}^{-1}$ for small Γ and χ) can still be done by the conjoining pressure. This is because in the conjoining case (unlike the disjoining one), work continues to be done even down to a very small film thickness.

### (b) Outlook

Remembering that the context for this work is waterflooding [23] and that the conjoining case can be achieved by suitably choosing salinity and/or ratio of divalent to monovalent ions to manipulate charge adsorption [49], it appears that salinity may indeed be a relevant process variable for waterflooding [36–44,46–48]. Based on the findings of the present work, process conditions for waterflooding if selected appropriately can ensure that the front of the droplet at least can be pushed along with less pressure. It may then be easier for the front of a droplet to enter a given capillary in the first place.

It is worth emphasizing however that we have considered the front of the droplet only. Moving the entirety of a droplet along a capillary requires consideration of both the front and rear. It is then possible that in waterflooding terms, a thicker film (disjoining case) [38,45,47] could still outperform a thinner one (conjoining case, as considered here). At the rear of the droplet, in the conjoining case at least, the expectation is that work would need to be done against the electro-osmotic conjoining pressure to peel the rear of the droplet away from the capillary wall. Viscous dissipation will still however be present.

Even in the classical uncharged case though [1], the situation at the rear of the droplet is more complicated to calculate, with the shape of the transition region becoming oscillatory [45,53,54]. Exactly how electro-osmotic conjoining effects will affect those oscillations at the rear of the droplet and how that will in turn impact on viscous dissipation is unclear. At the front of the droplet, in the presence of sufficiently strong conjoining pressures, multiple branches of solutions become possible (some of them being oscillatory and some not). In that case at least, what we found is that the more oscillatory solutions were associated with higher viscous dissipation, which in waterflooding terms would be undesirable.

## Data accessibility

This study used the same computation methodology as was employed already by the author in [49,50]. The dataset analysed is provided here as electronic supplementary material [75].
